# A framework for publishing primary biodiversity data

**DOI:** 10.1186/1471-2105-12-S15-I1

**Published:** 2011-12-15

**Authors:** Dave Roberts, Tom Moritz

**Affiliations:** 1 Zoology Microbiology Research Group, Zoology Department, Natural History Museum, SW7 5BD, London, UK; 2 1968½ South Shenandoah Street, Los Angeles, California 90034-1208, USA

## 

In modern science, substantial amounts of data are often synthesized into concise publications that present only certain facets of the information contained in the full dataset. Complete datasets are rarely available for independent study. A report published in 2008 [1] neatly summarized the inadequacy of this conventional approach and suggests the great potential for a new paradigm of data sharing:

"The digital age has presented the research community with new opportunities. Research findings in digital form can be easily moved around, duplicated, handed to others, worked on with new tools, merged with other data, divided up in new ways, stored in vast volumes and manipulated by supercomputers if their nature so demands. There is now widespread recognition that data are a valuable long-term resource and that sharing them and making them publicly available is essential if their potential value is to be realised.

There are two essential reasons for making research data publicly available: first, to make them part of the scholarly record that can be validated and tested; second, so that they can be re-used by others in new research."

We are now witnessing the emergence of new journals dedicated to the publication of data, specifically including publication of metadata that support well-informed retrieval of data from stable, persistent and secure sites.

Data sharing became a reality with the advent of computers capable of gathering large volumes of data concurrently with mechanisms to store and access those data. Taken together, these two factors gave us the technologies to move the data about, i.e. the world wide web [2]. The first significant instances of such data sharing occurred in the particle physics and astrophysics communities, joined soon by the molecular biology community. These early adopters, from 'big science' domains, used comparatively simple, standardized data structures with very large collections of data. The benefit of having large communities of scientists working on these massive datasets made clear the gains to be had from data sharing.

The science of biodiversity, by contrast, tends to collect data in much smaller quantities, handled in heterogeneous and non-standardized data structures. A US National Academy of Sciences [3] report noted that these datasets were "disaggregated components of an incipient network". More importantly, taxonomists and ecologists can still pursue their disciplines essentially as individuals or in small teams. The social structure of big machines and large collaborative teams has not yet made itself felt, although that is beginning to change [4]. In recent years a new discipline, 'biodiversity informatics', has gained prominence, focusing on discovery, integration, management and dissemination of data generated through biodiversity studies. Technical and infrastructural innovations have been achieved, but challenges remain. Biodiversity informatics must yet establish itself as scientifically, ecologically, socially and economically relevant and its long-term viability will depend on establishing that relevance. A major challenge has been the lack of a comprehensive 'data publishing framework' that can overcome technical, infrastructural, social, political, cultural and economic barriers. To address this, the Global Biodiversity Information Facility (GBIF) constituted a 'Data Publishing Framework Task Group' in the spring of 2009.

This special supplement presents the recommendations that emerged from the task group deliberations, together with papers that address additional challenges of data sharing in the biodiversity sciences. Specifically, we seek to examine the existing technical structures that can be used but, perhaps more importantly, we examine mechanisms to motivate data publication, through citation metrics and other measures of utility.

This supplement starts with an article by Moritz *et al. *[5] that summarizes the recommendations of the GBIF Data Publishing Framework Task Group. The remaining four articles focus on specific aspects that can help improve various components of the envisaged framework. Chavan and Penev [6] describe the 'data paper' as one of the incentivizing mechanisms for data publishing. Ingwersen and Chavan [7] postulate a 'Data Usage Index' as a metric to measure the impact of data publishing from usage patterns. Ariño *et al. *[8] conceptualize a 'Biodiversity Informatics Potential Index' to assess the potential of nations in furthering biodiversity informatics and to prioritize efforts to extend the data held. Finally, Goddard *et al. *[9] articulate the needs and obstacles of biodiversity data hosting infrastructure. It is clear that not all problems relating to data publishing have yet been resolved, and which of these proposed mechanisms will be taken up by the community involved in providing the data remains to be seen. The issue of persistent identifiers remains, and an acceptable community standard for data citation has yet to be established.

It is our hope that the approaches described here will stimulate a broad movement to publish biodiversity data, as is being demanded by funders in many countries. It will undoubtedly take time to establish a single default portal for biodiversity data that has a reputation similar to that GenBank has among molecular biologists.

## Competing interests

The authors declare that there are no competing interests.

## References

[B1] Research Information Network (RIN) To share or not to share: publication and quality assurance of research data outputs 2008 RIN http://www.rin.ac.uk/our-work/data-management-and-curation/share-or-not-share-research-data-outputs

[B2] Wikipedia: history of the world wide web http://en.wikipedia.org/wiki/History_of_the_World_Wide_Web

[B3] Esanu JM, Uhlir PF The role of scientific and technical data and information in the public domain: proceedings of a symposium 2003 Steering Committee on the role of Scientific and Technical Data and Information in the Public Domain, Office of International Scientific and Technical Information Programs Board on International Scientific Organizations, Policy and Global Affairs Division, National Research Council of the [US] National Academies http://www.nap.edu/openbook.php?isbn=030908850X 25057675

[B4] NEON and on Nature 2011 476 125 21833047 10.1038/476125b

[B5] Moritz T Krishnan S Roberts D Ingwersen P Agosti D Penev L Cockerill M Chavan V Towards mainstreaming of biodiversity data publishing: recommendations of the GBIF data publishing framework task group BMC Bioinformatics 2011 12 Suppl 15 S1 22373150 10.1186/1471-2105-12-S15-S1 PMC3287444

[B6] Chavan V Penev L Data paper: mechanism to incentivise discovery of biodiversity data resources BMC Bioinformatics 2011 12 Suppl 15 S2 22373175 10.1186/1471-2105-12-S15-S2 PMC3287445

[B7] Ingwersen P Chavan V Indicators for Data Usage Index (DUI): an incentive for publishing primary biodiversity data through global information infrastructure BMC Bioinformatics 2011 12 Suppl 15 S3 22373200 10.1186/1471-2105-12-S15-S3 PMC3287446

[B8] Ariño AH Chavan V King N Biodiversity Informatics Potential Index (BIP Index) BMC Bioinformatics 2011 12 Suppl 15 S4 22373233 10.1186/1471-2105-12-S15-S4 PMC3287447

[B9] Goddard A Cryer P Wilson N Data hosting infrastructure for primary biodiversity data BMC Bioinformatics 2011 12 Suppl 15 S5 22373257 10.1186/1471-2105-12-S15-S5 PMC3287448

